# Editorial: Therapeutic Process and Well-Being in Forensic Psychiatry and Prison

**DOI:** 10.3389/fpsyt.2020.626241

**Published:** 2020-12-22

**Authors:** Manuela Dudeck, Jürgen Leo Müller, Birgit Angela Völlm, Najat Khalifa

**Affiliations:** ^1^Department of Forensic Psychiatry and Psychotherapy, Ulm University, Ulm, Germany; ^2^Clinic for Psychiatry and Psychotherapy – Forensic Psychiatry, Human Medical Center Göttingen, Georg-August-University Göttingen, Göttingen, Germany; ^3^Department of Forensic Psychiatry, University of Rostock, Rostock, Germany; ^4^Department of Psychiatry, Queen's University, Kingston, ON, Canada

**Keywords:** forensic psychiatry, prison, therapy motivation, quality of life, risk reduction, risk assessment

Admission to secure forensic psychiatry or prison settings is accompanied by a massive loss of autonomy, freedom, and sense of control. A large proportion of residents in these institutions experience closed accommodation as a great burden, and many lose any hope for the future. This sense of hopelessness is reflected in the high suicide rates that are observed in secure forensic psychiatry and prison settings (1). In this book, 23 high quality studies are presented that delve into the complexities surrounding the therapeutic process and well-being in forensic psychiatry and prison settings. The issues addressed in the book are varied though equally pertinent, and span different international jurisdictions, therapeutic settings, and patient groups.

Büsselmann et al. studied the living conditions in 12 forensic psychiatric hospitals in Bavaria, Germany, and reported that creating a positive environment through supportive therapeutic rather than custodial interventions could reduce depressive symptoms and suicidal ideations among patients. Not all individuals suffer in the same way under the restrictive environment. As shown by Lutz et al., in the context of long-term imprisonment, inmates with a migration background are a particularly vulnerable group, and those who have few social relationships with fellow inmates are significantly more likely to experience psychological distress than native inmates. To investigate the highly regulated, secure, and prescriptive environments in forensic psychiatry settings, authors of two chapters in this book performed research on relevant measures: Tomlin et al. developed the Forensic Restrictiveness Questionnaire (FRQ), and Vorstenbosch and Castelletti evaluated the Forensic inpatient Quality of Life Questionnaire—Short Version (FQL-SV).

Protecting human rights is particularly important within the forensic psychiatry context because patients are not admitted voluntarily and so the treatment itself can be coercive in nature. Coercive measures (e.g., actions against the will of the patient, such as forced medication, seclusion, and restraint) represent an additional restriction of personal rights (2). Since the use of coercion in forensic psychiatric institutions remains controversial, additional empirical research is required to help understand the scale of the issue. In support of this endeavor, two studies in the present Research Topic contributed to the knowledge base by reporting on the rates of coercive measures: Flammer et al. analyzed the frequencies of seclusion, restraint, and compulsory administration of medications in all eight forensic facilities in the state of Baden-Wuerttemberg (Germany) in the years 2015 to 2017, and Lau et al. investigated coercive interventions in Switzerland's largest forensic hospital from 2010 to 2018. While performing coercive measures, mental health care professionals deal with complex ethical dilemmas that involve the principles of autonomy, justice, beneficence, and non-maleficence (3). Such dilemmas are even more prominent in forensic mental health care, where the restriction of personal rights is driven and legitimized not only by patient well-being but also by public safety interests. Because little is known about clinical ethics and the role of clinical ethics support in forensic mental health care in Germany, Franke et al. reported on the current structures and the availability and functioning of clinical ethics structures and identified specific ethics-related needs in forensic and general mental health care.

Another aim of the topic was to enhance the knowledge base on how to successfully promote patient motivation to engage in therapy even when the therapy is compulsory. Askola et al. explain that therapy in forensic psychiatric hospitals must not be limited to the treatment of the patient's mental illness. In a qualitative survey of forensic psychiatric nurses and patients, the authors found evidence that offense related therapeutic work, i.e., the analysis of the causes (e.g., stressors), evaluation of the emotional and situational characteristics, and development of possible prevention strategies, has a positive effect on the rehabilitation process. In a further study on therapy in forensic psychiatric hospitals, Bieg et al. examined the Therapeutic Cycles Model (4, 5). They were able to show that, contrary to the widely accepted view, the key therapeutic moments (referred to as “connecting”) in which change occurs are not accompanied by positive emotions but by feelings of discomfort or anxiety among patients. The authors came to this conclusion by analyzing transcripts of speech contributions of therapists and patients and assessing patients' well-being during therapeutic group sessions. Querengässer et al. focused on the causes of the high drop-out rates of patients with substance use disorders in forensic psychiatric hospitals. In Germany, around 50% of offenders with a substance use disorder terminate their therapy prematurely because of low prospects of success and are consequently sent back to prison (6). The authors studied the reasons for this high drop-out rate retrospectively from the perspective of both patients and therapists and found that the two groups had divergent views. They conclude that the inability to establish a common frame of reference for assessing the therapeutic process could be one of the main reasons for this high rate of therapeutic failure. The pharmacotherapeutic treatment of opiate-dependent offenders in German prisons was investigated by von Bernuth et al. Although the World Health Organization recommends opioid agonist treatment as a fundamental, evidence-based method in treating opioid dependence (7), only 52% of people who are dependent on opiates receive this treatment (8). In the study by von Bernuth et al., access to opioid agonist treatment appeared to be mainly dependent on initial receipt of this treatment at the time of imprisonment, detention duration, the prison in which an individual was detained, German nationality, and female sex.

Several articles in this research theme address the steps that can be taken to reduce re-offending rates after release from forensic psychiatric hospital or prison settings. In a feasibility randomized controlled trial, Khalifa et al. emphasized the importance of work but could not demonstrate any significant effects because the sample size was too small. Klinger et al. showed that positive long-term outcomes depend on the patients' social network. And McKendy and Ricciardelli investigated the factors that impede or support successful post-release outcomes in female prison inmates: notable differences were evident in relation to the presence of a mental disorder, the presence of substance addiction, and greater institutional adjustment (as indexed by institutional charges and segregation placements). To assist in treatment planning, risk monitoring, and decision-making, Hausam et al. incorporated measures of prison behavior into risk assessment and management procedures. By using a behavior rating scale, the group identified five inmate subtypes, i.e., Aggressive-Psychopathic, Asocial, Situational, Inconspicuous, and Inadequate-Dependent, with different predictive validity scores with regard to post-release recidivism. To establish relevant risk-need domains in sexual offenders, Eher et al. validated the Violence Risk Scale–Sexual Offense version (VRS-SO). The VRS-SO assesses criminogenic needs on the basis of three factors: sexual deviance, criminality, and treatment responsivity. It predicts sexual recidivism, as well as any new imprisonment or psychiatric placement. Wild et al. evaluated a treatment manual for the German therapy project “Prevention of Sexual Abuse” (9). This project provides treatment to patients with a self-reported sexual interest in children and adolescents, irrespective of whether or not they are pedophilic or have been prosecuted by the legal justice system. The results of the validation study provide indications for a relationship between treatment participation, reduced recidivism risk, and enhanced personal well-being of patients.

A high prevalence of mental disorders has been found among prisoners in several countries (10–13). Zhong et al. investigated psychiatric morbidity and comorbidity among female prisoners in China. Nearly two thirds of the sample fulfilled the criteria for at least one lifetime disorder according to the Diagnostic and Statistical Manual of Mental Disorders, fourth edition (DSM-4). The high level of psychiatric morbidity indicates unmet needs that require identification and treatment through therapeutic interventions in prisons. A simple-to-use tool to measure the severity of mental illness in correctional settings by mental health staff from different disciplines was developed by Jones et al. The authors adapted the severity scale of the Clinical Global Impression for use in correctional settings (CGI-C) and performed a reliability study.

Indirect or direct exposure to threats and violence and the perception of not being safe in an environment can be harmful to employees, too. Vogel et al. examined the correlations between misconduct in prison, a fundamental part of the everyday experience of correctional officers, and occupational factors such as team climate, job satisfaction, self-efficacy, and sick days. The results provide evidence for a positive association between rates of misconduct in prison and sick days and low self-efficacy. In a Canadian national online survey, Fusco et al. examined the views of public safety personnel. Correctional officers and forensic staff reported significantly more exposure to potentially psychologically traumatic events and higher rates of symptoms of mental disorders (including post-traumatic stress disorder, social anxiety, panic disorder, and depression) than wellness services employees.

Finally, Lebni et al. investigated the challenges facing women survivors of self-immolation. Although self-immolation accounts for only 1.6% of all burn cases treated in hospital in developed countries (14), it accounts for 16% of all cases in Iran. Beyond that, it accounts for more than 70% of suicides that result in death (15). Lebni et al. interviewed 19 women survivors and described a large number of problems as a consequence of self-immolation, ranging from psychological problems to a lack of social and legal support structures, incomplete treatment, poor self-care, and social problems. They conclude that reducing these women's problems and paving the way for their return to life requires multi-dimensional and community-based interventions.

## Author Contributions

MD wrote the first draft of the manuscript. JM, BV, and NK provided critical revision of the manuscript and important intellectual contributions. All authors read and approved the submitted version.

## Conflict of Interest

The authors declare that the research was conducted in the absence of any commercial or financial relationships that could be construed as a potential conflict of interest.
